# Synthesis of modified nanocomposite material and its use on removal of cesium from aqueous media

**DOI:** 10.3906/kim-2105-71

**Published:** 2021-08-24

**Authors:** Bilal ÇETİN, Mustafa ÖZCAN, Bektaş KARAKELLE

**Affiliations:** 1 Turkish Energy Nuclear and Mineral Agency, İstanbul, Turkey; 2 Department of Chemistry, Faculty of Science and Letters, İstanbul Technical University, İstanbul, Turkey

**Keywords:** Cs, Fe_3_O_4_, chitosan, adsorption, hexacyanoferrate, nanocomposite

## Abstract

A nanocomposite containing Fe_3_O_4_, chitosan (Ch), and hexacyanoferrate (HCF) was synthesized in the form of powder. The physicochemical properties of this nanocomposite are determined using different techniques including Fourier transform infrared spectroscopy (FTIR), X-ray diffraction (XRD), thermogravimetric analysis (TGA), scanning electron microscopy (SEM). The existence of Cs (Cs) ions onto the surface of the nanocomposite was verified by dispersive X-ray spectroscopy (EDX). FTIR spectra confirmed that the nanocomposite was well coordinated. The batch technique was applied to evaluate the influences of initial pH value, temperature, contact time, shaking rate, initial Cs concentration, and competing cations on the efficiency of Cs removal. The maximum adsorption capacity for Cs ions of nanocomposite was determined as 34.36 mg/g at the initial pH = 5 of the aqueous solution. The equilibrium data fitted well the linearized Langmuir isotherm equation, which has the higher correlation coefficient (0.999). Thermodynamic parameters such as free energy (ΔG°), enthalpy (ΔH°), and entropy (ΔS°) indicated that the adsorption was exothermic and not spontaneous.

## 1. Introduction

Radioactive waste (RW) is an inevitable residue from the use of radioactive materials (RMs) in industry and the medical sector, as well as from research and nuclear establishments. The management and disposal of such RW is, therefore, an issue relevant to almost all countries. Also, the development of nuclear science and technology, in particular the wide application of nuclear power, seriously threatens the human environment through radioactivity contamination. Two of the most important fission radionuclides from the reactor, ^137^Cs and ^134^Cs, are considered potentially dangerous to human health and to the environment because the relatively high yield in nuclear power plants, long half lives, and high solubility of Cs (Cs) can cause its migration through groundwater to the biosphere [1]. In other respects, they can be easily adsorbed by terrestrial and aquatic organisms, because of their chemical similarity to potassium (K). The formation of complexes does not have a significant effect on Cs speciation, and the predominant aqueous species in groundwater is the free Cs ion [2]. Different techniques, such as solvent extraction, evaporation, and ion exchange, are usually used for the treatment of aqueous waste solutions containing Cs ion [3–4].

Recently, considerable attention has been paid to the synthesis of nanocrystalline materials (NMs) to increase their industrial applications, owing to their large surface area and shorter diffusion paths compared to the micro sized crystals (MSC) [5]. In spite of its benefits, there is a problem related to the separation of spent nanomaterials from the aqueous medium. The technique for the former’s separation involves the use of centrifugation process, which is very costly and time consuming. Lately, researchers proceeded to use the magnetic separation technique (MST) since it is efficient, simple, and fast [6]. Magnetic nanoparticles (MNs), which are composed of a MN based core and a functional shell that can adsorb contaminants, such as heavy metals or radioactive nuclides have been intensively analyzed for environmental remediation applications due to their high surface areas and ability to be magnetically collected using an external magnet [7,8]. Fe_3_O_4_ has low toxicity and is small in size. Magnetization of adsorbent matrix facilitates separation of the used sorbent [9].

Chitosan (Ch) is a biopolymer obtained by the deacetylation of chitin [10–12]. Moreover, metal nanoparticles can be incorporated with Ch to form a composite [13]. Ch has an excellent adsorption capacity for various metals, such as Cr(VI), Cu(II), Pt(IV), Pd(II), Co(II), Ni(II), and Fe(III). Metal uptake by Ch is primarily attributed to the hydroxyl and amine groups existing in the polymer chain, the functional groups can react with the different metallic species through the mechanism of chelation and/or ion exchange [14]. Ch has two hydroxyl groups and one amino group on each glucosamine monomer, the amino groups can be strongly linked to metal ions, and amino and hydroxyl groups can be interactive with organic compounds via hydrogen bonding. At the same time, the amino group of Ch is easily protonated in acidic solutions, restricting the application in the adsorption process [15].

Different sorbents modified with Ch have been used for heavy metal removal such as chitosan-tripolyphosphate beads, chitosan-cellulose hydrogel beads, chitosan-graft-γ-cyclodextrin,, tetraethylenepentamine modified magnetic chitosan resin, Guar Gum/Magnetite/Chitosan, magnetic Schiff’s base chitosan composite, chitosan-clay, chitosan-inorganics etc. Rough range of metal sorption capacity by chitosan-based sorbents have been varied between 4.1 mg/g and 3170 mg/g for heavy metal removal [16–19].

Metal hexacyanoferrate (MHCF) analogues are the inorganic complexes known for their versatile applications [20]. Transition metal hexacyanometallates (TMHCM) usually have an open channel framework appropriate for small molecules separation, and their crystal structure is closely related to the coordination adopted by the metal centers. In hexacyanometallates (HCMs), the involved transition metals (TM) are usually found with octahedral coordination within the cubic unit cell (Fm-3m). Some zinc hexacyanoferrates (ZnHCF) have been reported as hexagonal where Zn^2+^ atom is found tetrahedrally coordinated to four nitrogen (N) atoms from cyano (CN) ligands. Such coordination provides a relatively high thermal stability to these materials and also an interesting porous framework because both metal centers have saturated their coordination sphere with atoms from the bridge group (−CRN−) [21].

In the present work, Fe_3_O_4_ was synthesized from iron ore waste and used to form the nucleus of the nanocomposite adsorbent. Later, nanocomposite containing chitosan and metal hexacyanoferrate was synthesized using Fe_3_O_4_. The synthesized adsorbent was characterized and used to determine optimum adsorption parameters such as initial pH value, temperature, contact time, shaking rate, initial Cs concentration, adsorbent amount. The results obtained were used to determine the adsorption type, isotherms, adsorption capacity, and thermodynamic values.

## 2. Experimental

### 2.1. Materials

Iron ore waste used in obtaining of Fe_3_O_4_ was supplied by Kroman Çelik Sanayii A.S. (Istanbul, Turkey). The chitosan compound was purchased from İsmail Aslan Chemistry (Sakarya, Turkey), K_4_Fe(CN)_6_.3H_2_O (HCF) was purchased from Merck KGaA (Darmstadt, Germany), other chemicals are analytical grade and used as received without additional processing.

### 2.2. Preparation of composite

#### 2.2.1. Synthesis of magnetic nanoparticles Fe_3_O_4_

A total of 25 g of iron ore waste was taken and treated with 90 mL of HCl acid at 90 °C for 6 h. Insoluble particles filtered from the mixture were brought to room temperature by means of blue band filter paper, and this filtrate containing Fe (III) was diluted to 250 mL (Stock A) and maintained under pH = 1. A total of 100 mL of stock a solution was taken and Fe (III) was reduced to Fe^0^ in the form of black iron particles by mixing with NaBH_4_ in an ice-cold water bath quickly. NaBH_4_ was added until the solution became colorless. The solution containing metallic iron was filtered through blue band filter paper to obtain black iron particles, first washed with ethanol and then with distilled water twice. The resulting metallic iron was added to 100 mL of Stock A solution and a Fe (II) solution (Stock B) was obtained. Fe amount in Stock A and Stock B was determined by ICP-OES (Perkin Elmer-Optima 8000). Aliquots were taken from stock A and Stock B solutions with a Fe^3+^/Fe^2+^ molar ratio of 2:1 and placed an erlenmeyer. 25% NH_3_ was added at room temperature until the mixture had a pH value of 10, and then Fe_3_O_4_ was precipitated. The mixture was filtered after the magnetic properties of the compound obtained are checked by an external magnet. Fe_3_O_4_ was washed three times with pure water and dried for one day at 60 °C in the oven.

#### 2.2.2. Synthesis of Fe_3_O_4_/Chitosan composite

0.6 g chitosan was dissolved in 15 mL (2% v/v) acetic acid with vigorous stirring for 1 h. Aliquots from Stock A and Stock B solutions were taken with a Fe^3+^/Fe^2+^ molar ratio of 2:1 and added to the mixture containing chitosan. The entire mixture was stirred for 2 h until a dark red viscous mixture was obtained. The above-mentioned mixture was added to 1.3 M 50 mL NaOH solution and stirred for 12 h at room temperature. The precipitate formed was filtered and washed with distilled water. All these processes were carried out in an inert N_2_ atmosphere to prevent Fe^2+^ oxidation. The precipitate was dispersed in 30 mL (20% w/v) sodium tripolyphosphate (STPP) solution and mixed for 2 h. The mixture was filtered, and the precipitate was washed in distilled water and ethanol, respectively, and then dried at 60 °C for one day. The composite shows whether the magnetic properties were checked with the help of an external magnet.

#### 2.2.3. Synthesis of Fe_3_O_4_/Ch/HCF composite

Fe_3_O_4_/Chitosan was added to 0.1 M K_4_Fe(CN)_6_.3H_2_O solution and stirred for 3 h in N_2_ atmosphere. Then, 0.1 M ZnCl_2_ was slowly added dropwise in an inert N_2_ atmosphere and stirred for 3 h. The precipitate was filtered and recrystallized in a 3:2 isopropyl alcohol-acetone mixture, then washed 3–4 times with distilled water and allowed to dry at room temperature. After the composite obtained was pulverized, it was sieved in a 38 micron-sized sieve and kept in an airtight container.

### 2.3. Adsorption experiments

The batch technique was used to adsorb Cs onto nanocomposite adsorbent. A total of 100 mg/L Cs^+^ stock solution prepared, and adsorption experiments were carried out in Memmert-WNB 29 model temperature-controlled shaking water bath by taking aliquots from this stock solution. pH studies between 2 and 10, temperature studies between 25 °C and 60 °C, adsorbent amount studies between 10 mg and 100 mg, contact time studies between 30 min and 300 min, shaking rate studies between 80 rpm and 180 rpm, initial Cs concentration studies between 2.5 mg/L and 80 mg/L were performed. After the adsorption experiments, the Cs concentration in the supernatant obtained was determined by the flamed atomic emission spectrometer (Varian Spectr AA 200, 852.7 nm wavelength, slit: 1.0 nm, 2000 mg/L Potassium (K) ionization suppressor) with air/acetylene. The adsorption capacity q_e_ (mg/g) in equilibrium was calculated using the following equation:


(1) $$q_{e}=\frac{(C_{0}-C_{e})V}{W}$$

where C_0_; Initial concentration (mg/L), C_e_; Concentration in equilibrium (mg/L), V; Solution volume (L), W; adsorbent weight (g).

Jeol brand JSM 639OLV model scanning electron microscope (SEM) for determining the morphology of Fe_3_O_4_/Ch/HCF composite, Quantachrome brand Nova 2200e model BET analyzer for determining the surface area, Olympus brand Delta XRF device for determining the Fe amount in the different iron ore wastes were used and Netzsch Brand STA 449C model device was used for DTA and TGA.

## 3. Results and discussion

### 3.1. Characterization

#### 3.1.1. SEM images

**Figure 1:**
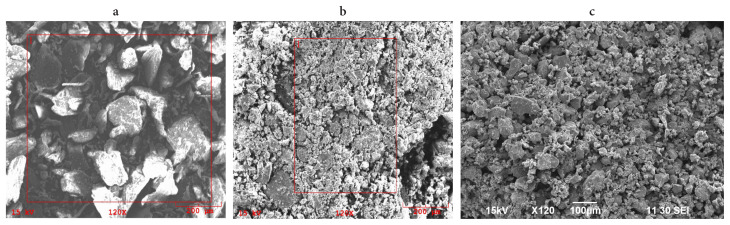
SEM image of (a) pure chitosan, (b) Fe_3_O_4_/Ch and (c) Fe_3_O_4_/Ch/HCF nanocomposite.

**Figure 2:**
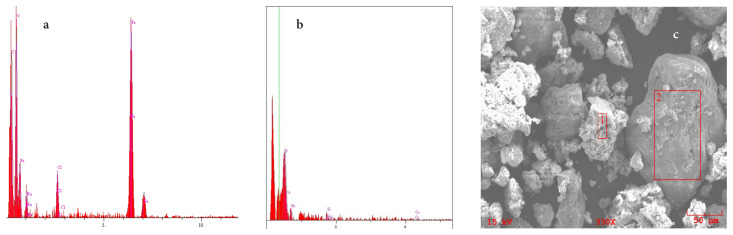
EDX graph of Fe_3_O_4_/Ch/HCF nanocomposite (a) before Cs adsorption and (b) after Cs adsorption and (c) SEM image of Cs adsorbed Fe_3_O_4_/Ch/HCF nanocomposite.

SEM image of pure chitosan, Fe_3_O_4_/Ch and Fe_3_O_4_/Ch/HCF composite is given in Figure 1. As can be seen from the image, nanocomposite does not have a specific shape and the size of the particles varies between 1 μm and 25 μm. EDX analysis (Figure 2) was performed to determine the elemental composition of composite and whether Cs adsorption was performed, and the results are presented in Table 1. There is no Cs in the structure of the composite before adsorption, but, when the results of EDX spectrum after the adsorption are examined, the presence of Cs is determined, and these data prove that Cs adsorption occurs on the composite.

#### 3.1.2. BET and XRD diffraction

**Figure 3:**
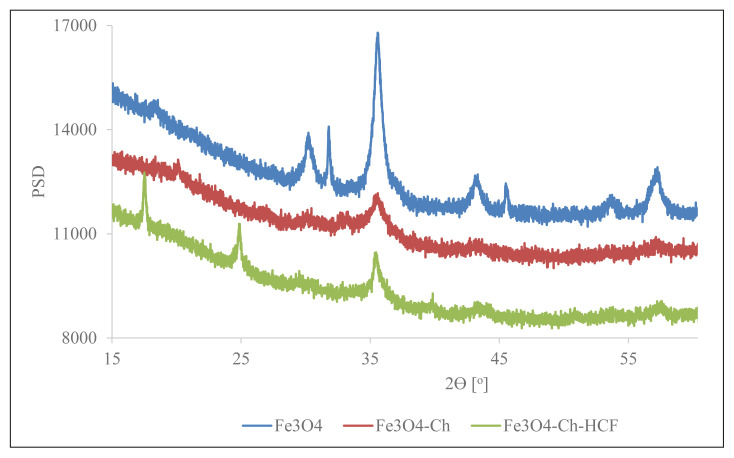
Fe_3_O_4_, Fe_3_O_4_/Ch and Fe_3_O_4_/Ch/HCF XRD patterns.

As a result of the BET analysis, the surface area of 1 g of the composite was determined as 60.87 m^2^. XRD analysis was carried out to determine the crystal size of the composite and to determine Fe_3_O_4_, Chitosan, and HCF in its structure. XRD patterns are shown in Figure 3. Deby–Scherrer equation used in calculating the crystal size of materials is expressed as follows:


(2) $$L=\frac{K\lambda }{\beta cos\theta }$$

where L is the average crystal size, K is the constant about crystal shape, λ is the X-ray wavelength (nm), β is the peak width of the diffraction peak profile at half maximum height due to the small crystal size (rad), and θ is half of the Bragg Angle.

In the calculation made using the Deby-Scherrer equation, the particle size of Fe_3_O_4_, which composes the composite, has been calculated as 12.315 nm, and it has been determined that the composite is nanocomposite because it is a nano-sized component. When the XRD patterns of Fe_3_O_4_, Fe_3_O_4_/Chitosan, and Fe_3_O_4_/Ch/HCF are compared to each other, similar peaks are seen at 35, 56, and 63 2⊝ positions, which proves to us that there is Fe_3_O_4_ in the structure of the nanocomposite. Since there are no sharp peaks in the XRD graph of chitosan, it is not possible to compare it with the XRD graph of nanocomposite.

#### 3.1.3. FTIR spectrum

**Figure 4:**
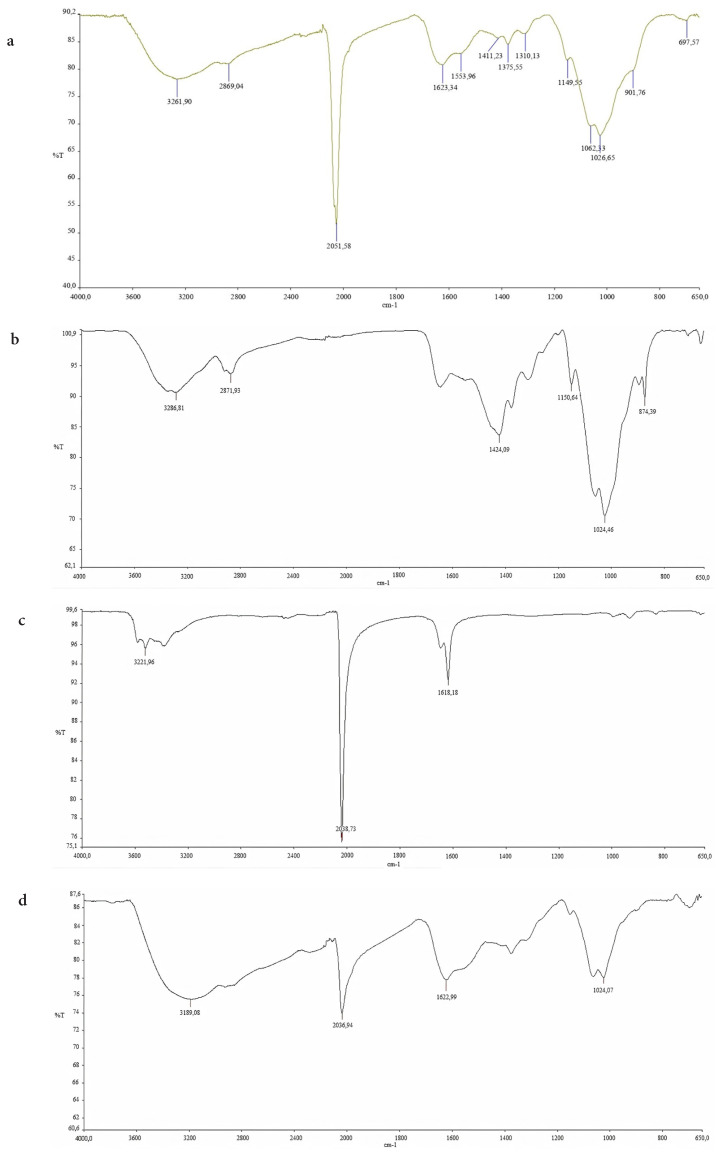
FTIR spectrum of (a) Fe_3_O_4_/Ch/HCF nanocomposite, (b) Chitosan and (c) HCF and (d) Cs adsorbed-Fe_3_O_4_/Ch/HCF.

FTIR spectrum of nanocomposite, Chitosan and HCF is given in Figure 4. The peak at 2051 cm^−1^ corresponds to the functional group −C≡N in the structure K_4_[Fe(CN)_6_] and easily can be seen from the FTIR spectrum of pure HCF. It is understood from the graph that this peak is quite sharp and prominent. Therefore, the presence of K_4_[Fe(CN)_6_] in the structure of the nanocomposite material has been proven. In addition, 1623 cm^−1^, 1553 cm^−−^ peaks in the structure of Chitosan correspond to N-Acetyl group, 1149 cm^−1^ peak corresponds to C-O-C bridge, 1062 cm^−1^ and 1026 cm^−1^ peaks correspond to C-O stretching and 3261 cm^−1^ peak corresponds to NH, OH, and intermolecular H bonds. All of abovementioned peaks related to Chitosan from the FTIR spectrum of nanocomposite can be easily seen from the FTIR spectrum of pure Chitosan, and all of these abovementioned peaks are proof of the presence of chitosan in the nanocomposite. When we examine the FTIR spectrum of Cs adsorbed-Fe_3_O_4_/Ch/HCF nanocomposite the peak at 2051 cm^−1^ in corresponds to the functional group −C≡N is shifted to 2036 cm^−1^ as well as 3261 cm^−1^ peak corresponds to NH is shifted to 3189 cm^−1^. These observations show that NH and −C≡N functional groups of nanocomposites play significant role on Cs adsorption on nanocomposite.

#### 3.1.4. Thermal curves

**Figure 5:**
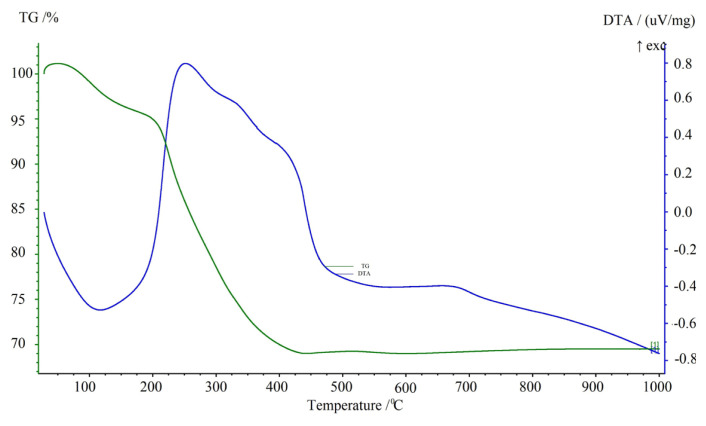
TGA (Green line) and DTA (Blue line) plot of Fe_3_O_4_/Ch/HCF nanocomposite and chitosan.

TGA and DTA plots of Fe_3_O_4_/Ch/ HCF nanocomposite are shown in Figure 5. According to the DTA result given for chitosan in the literature, it was stated that the decaying peak of the chitosan is exothermic and occurs between 270–337 °C. The reason for this has been shown to cause thermal degradation of amino and N-acetyl groups [22]. When Fe_3_O_4_/ chitosan nanocomposite was heated up to 200 °C at room temperature, its mass decreased as a result of evaporation of the adsorbed water in the nanocomposite. Mass loss between 200 °C and 400 °C is thought to result from the degradation of chitosan cross-links in the nanocomposite [23]. When the results with TGA and DTA were compared with the literature, it was understood that the mass loss was experienced due to the removal of the adsorbed water molecules between 0 and 200 °C and the decay of chitosan between 200 and 400 °C. It is determined that the DTA curve is endothermic between 0 and 200 °C, and it is caused by degraded water molecules, and DTA curve is exothermic between 200–400 °C because of the degradation of chitosan.

### 3.2. Adsorption experiment

#### 3.2.1. Effect of initial pH value

**Figure 6:**
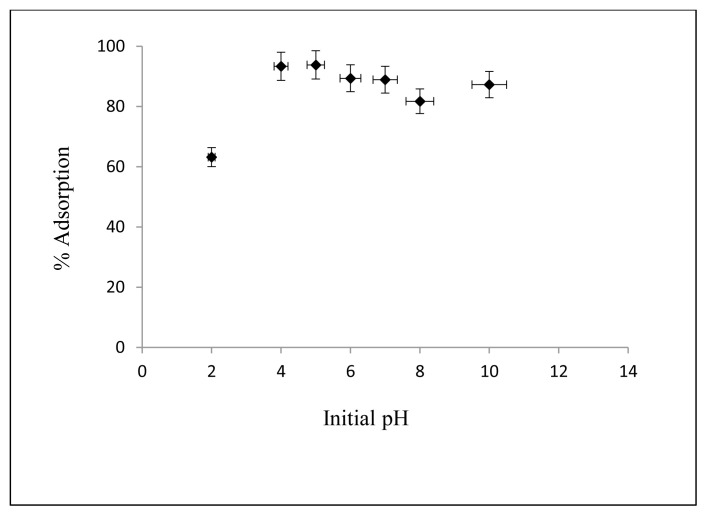
Effect of the initial pH value on Cs adsorption.

Effect of initial pH on adsorption of Cs on nanocomposite is shown in Figure 6. The concentration of Cs is 2.5 mg/L, Cs solution amount is 50 mL, the amount of adsorbent is 10 mg, the temperature is 25 °C, the contact time is 30 minutes, the shaking rate is kept at 125 rpm, and initial pH values are changed between 2, 4, 6, 7, 8 and 10. As can be seen in Figure 6, an increase in adsorption between pH = 2 and pH = 4 has been observed and reached the maximum value, no significant pH value changes between 4 and 10 have been observed. Therefore, the optimum initial pH value was chosen as 5, which is the pH value of the solution.

#### 3.2.2. Effect of temperature

**Figure 7:**
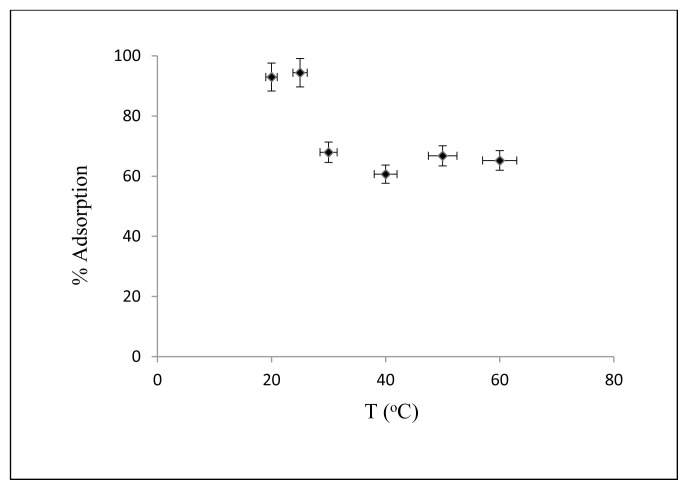
Effect of temperature on Cs adsorption.

The temperature effect on adsorption of Cs on nanocomposite is shown in Figure 7. The concentration of Cs is 2.5 mg/L, Cs solution amount is 50 mL, initial pH = 5, the amount of adsorbent is 10 mg, the contact time is 30 minutes, the shaking rate is kept at 125 rpm the temperature values are changed between 25, 30, 40, 50 and 60 °C. As can be seen from Figure 7, the maximum adsorption occurred at 25 °C.

#### 3.2.3. Effect of adsorbent amount

**Figure 8:**
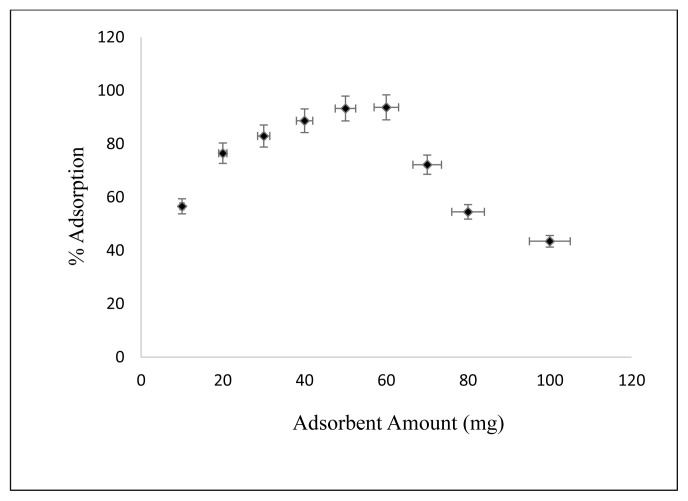
Effect of adsorbent amount on Cs adsorption.

The effect of adsorbent amount on adsorption of Cs on nanocomposite is shown in Figure 8. Cs concentration is 2.5 mg/L, initial pH: 5, Cs solution amount is 50 mL, the temperature is 25 °C, contact time is 30 min, shaking rate is kept at 125 rpm and adsorbent amounts are changed 10, 20, 30, 40, 50, 70, and 100 mg, respectively. As can be seen from Figure 8, the maximum adsorption occurred at 50 mg.

#### 3.2.4. Effect of contact time

**Figure 9:**
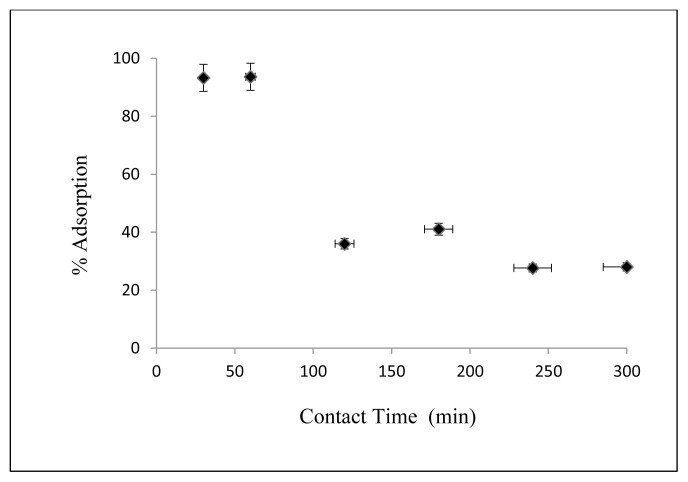
Effect of contact time on Cs adsorption.

The effect of contact time on the adsorption of Cs on nanocomposite is shown in Figure 9. Cs concentration is 2.5 mg/L, Cs solution amount is 50 mL, initial pH = 5, temperature is 25 °C, the adsorbent amount is 50 mg, shaking rate is kept at 125 rpm and contact times are changed 30, 60, 120, 180, 240, and 300 min respectively. As can be seen from Figure 9, the maximum adsorption occurred in 30 minutes.

#### 3.2.5. Effect of initial Cs concentration

**Figure 10:**
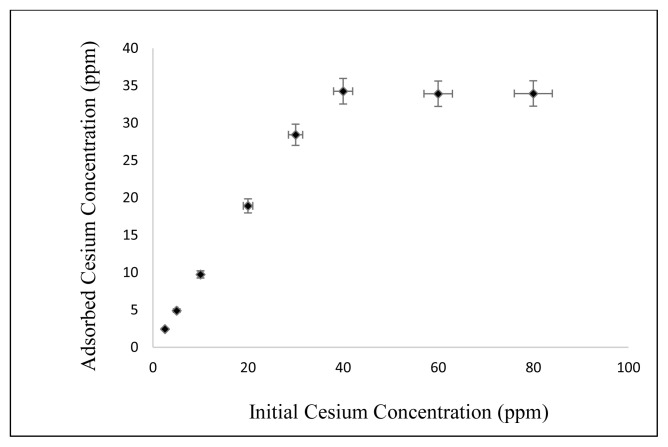
Effect of initial Cs concentration on Cs adsorption.

The effect of initial Cs concentration on adsorption of Cs on nanocomposite is shown in Figure 10. Temperature is 25 °C, Cs solution amount is 50 mL, initial pH = 5, adsorbent amount is 50 mg, contact time is 30 min, shaking rate is kept at 125 rpm and initial Cs concentration values changed 2.5, 5, 10, 20, 30, 40, 60, and 80 mg/L, respectively. As can be seen from Figure 10, the maximum adsorption occurred at a concentration of 40 mg/L Cs.

#### 3.2.6. Effect of shaking rate

**Figure 11:**
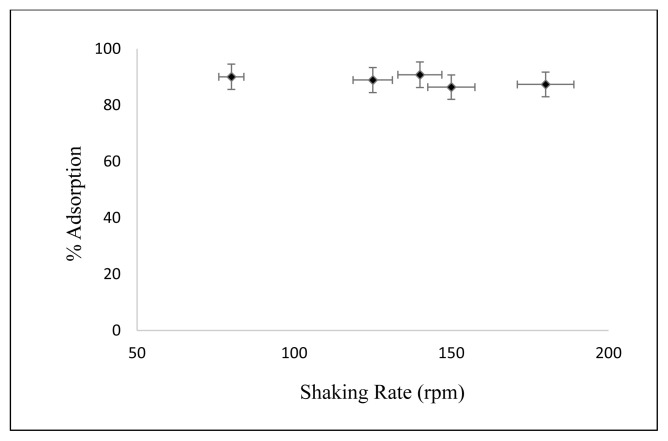
Effect of shaking rate on Cs adsorption.

The effect of shaking rate on adsorption of Cs on nanocomposite is shown in Figure 11. The concentration of Cs is 2.5 mg/L, Cs solution amount is 50 mL, initial pH = 5, the temperature is 25 °C, the amount of adsorbent is 50 mg, the contact time is kept constant in 30 min, and the shaking rate values are changed 80, 125, 140, 150, and 180 rpm, respectively. As understood from Figure 11, the maximum adsorption occurred at 140 rpm.

#### 3.2.7. Adsorption isotherms

Analysis of the equilibrium relationship between adsorbed Cs ions and the adsorbent and the balance data obtained from the experiments is very important in elucidating the adsorption properties of Fe_3_O_4_/Ch/HCF nanocomposite adsorbent. Adsorption equilibrium data obtained at different Cs concentrations (2.5–80 mg/L) and at different temperatures (25–60 °C) were applied in the Langmuir and Freundlich isotherm models, and the equations for these isotherms are expressed, respectively, as follows:


(3) $$q_{e}=\frac{q_{max}K_{l}C_{e}}{1+K_{l}C_{e}}$$

(4) qe=KfCe1n

where C_e_ is equilibrium concentration of Cs in solution (mg/L), q_e_ is adsorption capacity of adsorbent in equilibrium (mg/g) q_max_ is adsorbent’s maximum adsorption capacity, K_l_ is Langmuir equilibrium constant (L/mg), K_f_ is Freundlich equilibrium constant (L/mg).

Graphs of Freundlich and Langmuir isotherms obtained from cesium adsorption balance data are given in Figure S1. and Figure S2. at the supplementary materials, respectively. The maximum adsorption capacities, error values calculated by sum of squared error (SSE), and adsorption constants obtained from Freundlich and Langmuir isotherm models in Cs adsorption on nanocomposite are presented in Table 2. As seen in Table 2, when the correlation coefficient values (R^2^) of Cs ions on adsorption of Fe_3_O_4_/Ch/HCF on nanocomposite adsorbent and error values (SSE) were examined, Langmuir isotherm model was observed to fit better than Freundlich model and maximum adsorption capacity was determined as 34.36 mg/g.

As a result of fit to the Langmuir isotherm model of the adsorption of Cs ions on Fe_3_O_4_/Ch/HCF nanocomposite adsorbent, It can be concluded that the adsorption surface is homogeneous and adsorption occurs in a single layer on the surface, the adsorption is localized and the adsorbed ions do not move on the surface, the adsorption enthalpy is independent from the surface coating, there are no interactions between adsorbed ions, and the amount of substance adsorbed per unit surface has no effect on the adsorption rate.

#### 3.2.8. Adsorption mechanism

It is stated that chitosan interacted with iron ions and hydroxyl groups on the surface of Fe_3_O_4_ by means of functional amino groups. The iron oxide nuclei form iron oxide aggregates and iron oxide polycrystalline nanostructures are formed on the surface of these aggregates by self-assembly; thus, it was concluded that the synthesized structure is in the form of mesopore structure [24]. Amino groups in the structure of chitosan are protonated as -NH^3+^ in acidic environments, and chitosan has a good chelating feature, because of the presence of N atom in amino groups that can be well-coordinated with ions with empty orbitals such as Fe^2+^ and Fe^3+^ [25]. It has been stated that chitosan is polycationic when dissolved in acid and it presents −NH^3+^ regions. Sodium tripolyphosphate (Na_5_P_3_O_10_) is mentioned that when it dissolves in water, it decomposes into hydroxyl and phosphoric ions. It has been reported that at high pH values, −OH− and P_3_0_5_^−^ are formed and these ions compete with each other in interaction with the −NH^3+^ regions, and, as a result of deprotanization of −OH^−^ ions, P_3_0_5_^−^ ions interact with the −NH^3+^ groups of chitosan by ionic cross-linking [26]. Fe_3_O_4_/Chitosan formation; It is stated that Fe^3+^ ions form chelates on the chitosan chain through amino and hydroxyl groups on chitosan, and the chelating effect of the −NH_2_ and −OH groups distributes iron ions homogeneously [23]. Also, the FTIR spectrum of Cs adsorbed- Fe_3_O_4_/ Ch/HCF nanocomposite proves that −C≡N in the structure of K_4_[Fe(CN)_6_] and −NH_2_ the structure of Chitosan plays a significant role in the adsorption of Cs onto nanocomposite.

#### 3.2.9. Thermodynamics of sorption

Adsorption thermodynamics were investigated to further illuminate the effect of temperature on energy changes in Cs adsorption and adsorption process. Changes in adsorption capacity caused by temperature change can be explained based on thermodynamic parameters such as free energy (ΔG°), enthalpy (ΔH°) and entropy (ΔS°). Thermodynamic equations are expressed as follows


(5) $$\Delta G{}^{\circ}=-RT(lnK_{e}^{0})$$

(6) $$\text{ln}(\frac{K_{L}}{\gamma_{e}})=-\frac{\Delta \text{H}{}^{\circ}}{R}\frac{1}{T}+\frac{\Delta \text{S}{}^{\circ}}{R}$$

(7) ΔG°=ΔH°-TΔS°

Here, R is ideal gas constant (8.314 j/molK), T is adsorption temperature (K), *K_l_* is the Langmuir isotherm constant, *Y_e_* is the coefficient of activity for Cs and $K_{e}^{0}$ is the dimensionless thermodynamic equilibrium constant and can be calculated by the equation given below [27]:


(8) $$K_{e}^{0}=\frac{1000.k_{l}.Molecular\;weight\;of\;adsorbate.{\left[adsorbate\right]}^{0}}{\gamma }=\frac{K_{e}}{\gamma }$$

Enthalpy (ΔH°) and entropy (ΔS°) and then free energy (ΔG°) values were calculated from the graph equation obtained by drawing 1/T (K^−1^) values against ln $K_{e}^{0}$ values obtained from temperature effect studies on Cs adsorption. ΔH^0^ value was calculated −21.50 kj/mol, ΔS° value was calculated 0.081 kj/molK, ΔG° values at 25 °C, 40 °C and 60 °C were calculated as −45.59, −46.80 and −48.42 kj/mol, respectively. The adsorption enthalpy (ΔH°) is negative, and the entropy (ΔS°) is positive; due to this reason, the adsorption process is exothermic and spontaneous. The fact that the adsorption enthalpy (ΔH°) is negative and entropy (ΔS°) are positive as well as the free energy (ΔG°) value is also negative indicates that the adsorption occurs spontaneously at all temperatures. Additionally, Cs adsorption onto nanocomposite adsorbent (Fe_3_O_4_/ Ch/HCF) is occurred physically due to low adsorption enthalpy (ΔH°) which is −21.50 kj/mol [28].

#### 3.2.10. The effect of competing ions

The effect of competitive ions (Sr^2+^, Co^2+^) on Cs adsorption was investigated. Sr^2+^ and Co^2+^ ions have been selected because radioactive strontium and cobalt isotopes are often found together with the radioactive isotope of Cs in liquid radioactive waste arised from operations, decommissioning, and accidents of reactors. In the aspect of radiological safety, strontium (Sr-90), cobalt Co-60), and Cs (Cs-137) are major radioactive isotopes because of their relatively long half-lives, high solubility, and transferability [14]. A total of 50 mL solution was prepared in which each ion had a concentration of 40 mg/L. As it can be seen in Table 3, when there is no competitive ion in the solution, adsorption is 85.65%, while this value has decreased to 65.53% in the presence of competitive ions. This may be due to the fact that foreign ions are adsorbed to the surface of the nanocomposite by complexity or nonspecific surface interactions rather than the ion exchange process. Table 3 also shows that Sr^2+^ or Co^2+^ effects, which are separate from each other on Cs adsorption, is almost same.

#### 3.2.11. Desorption studies

Loaded nanocomposites obtained from earlier studies were used to determine the desorption behavior of Cs^+^. Deionised (DI) water, 0.1 M, 0.5 M, and 1 M HCl and 0.1 M, 0.5 M, and 1 M NaOH were used as desorption agents. Loaded nanocomposites (0.05 g each) were transferred to a clean flask containing 50 mL of 0.1 M, 0.5 M, and 1 M HCl, DI water and 0.1 M, 0.5 M, and 1 M NaOH. The mixture was agitated in a rotary shaker at 140 rpm for 30 min. The desorption efficiency is determined by Eq. (9):


$$Desorption\;\%=\frac{amount\;of\;metal\;ion\;desorbed}{amount\;of\;metal\;ion\;adsorbed}\times 100$$

**Figure 12:**
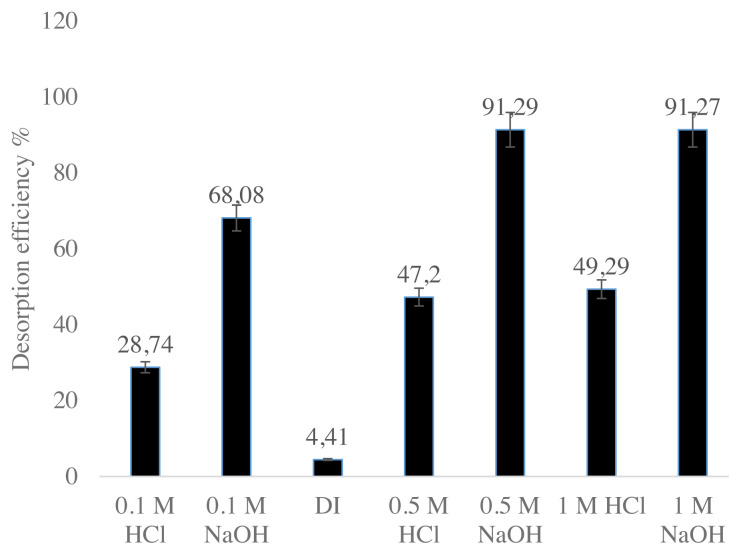
Desorption studies of nanocomposite with Cs^+^ ions.

Figure 12 shows the result of the desorption experiments. NaOH was most effective, causing 91.29% of the adsorbed Cs to desorp. 0.5 M NaOH and 1 M NaOH showed almost same desorption effect. The results suggest that the loaded nanocomposite may be suitable for disposal into designated landfill if it is protected from alkaline leachate [29].

## 4. Conclusion

Fe_3_O_4_/Ch/HCF nanocomposite was synthesized using Fe_3_O_4_ obtained using iron ore waste. Temperature, pH value, agitation rate, contact time, adsorbent amount and initial Cs concentration were determined 25 °C, pH: 5, 140 rpm, 30 min, 0.05 g adsorbent and 40 mg/L Cs^+^, respectively. The presence of competitive ions led to a decrease in the amount of Cs adsorbed on nanocomposite, which is due to complexation and nonspecific surface interactions. According to the information obtained from studies with Fe_3_O_4_, chitosan and hexacyanoferrates in the literature, iron ions in Fe_3_O_4_ interact with amino and hydroxyl groups on chitosan and form a core-shell structure with coordination bonds, STPP dissolves in water and forms OH^−^ and P_3_0_5_^−^ ions, and, after that, these ions interact with amine groups of chitosan. It is thought that OH^−^ and P_3_0_5_^−^, which are formed as a result of the decomposition of STPP and −CN groups in the structure of hexacyanoferrate, interact with iron ions (Fe^2+^ and Fe^3+^) and keep hexacyanoferrates attached to the core-shell Fe_3_O_4_-chitosan composite. It was determined that the adsorption fits the Langmuir isotherm model better and the maximum adsorption capacity is 34.36 mg/g. As the result of the calculation of the thermodynamic parameters, the adsorption enthalpy (ΔH°) is negative, and entropy (ΔS°) is positive as well as the free energy (ΔG°) value is also negative indicates that the adsorption occurs spontaneously at all temperatures.

In the literature, adsorption capacity of various sorbent used on Cs removal is ranging between 6.68 mg g^−1^ and 306 mg g^−1^ and illustrated in the table [29–33]; therefore, Fe_3_O_4_/Ch/HCF nanocomposite has been used effectively in the removal of Cs from the aqueous medium as well as the reuse of the waste generated in the iron industry. The adsorption process with this nanocomposite can be used as a fast and effective method.

Figure S1 Freundlich isotherm graph obtained from cesium adsorption

Figure S2 Langmuir isotherm graph obtained from cesium adsorption

## Figures and Tables

**Table 1:** EDX result of Fe_3_O_4_/Ch/HCF nanocomposite before and after Cs adsorption.

Elt.	Line		Intensity (c/s)	Error 2-sig	Conc.	Units
C	Ka	**Before Adsorption**	18.44	0.86	26.18	wt.%
		**After Adsorption**	7.00	0.53	12.54	wt.%
O	Ka	**Before Adsorption**	19.73	0.89	21.12	wt.%
		**After Adsorption**	21.13	0.92	22.84	wt.%
Fe	Ka	**Before Adsorption**	35.32	1.19	47.62	wt.%
		**After Adsorption**	36.02	1.20	62.43	wt.%
Cs	La	**Before Adsorption**	-	-	-	wt.%
		**After Adsorption**	0.61	0.16	0.98	wt.%

**Table 2:** Comparison of Freundlich and Langmuir constants.

Model	Adsorbent	Cs(I)			
Langmuir	Fe_3_O_4_/Ch/HCF	**q**	**K** **_L_**	**R** **^2^**	**SSE**
		3	2.01	0.999	36.34
Freundlich		**K**	**1/n**	**R** **^2^**	**SSE**
		1	0.363	0.831	769.28

**Table 3:** Sr^2+^ and Co^2+^ effect on Cs adsorption.

Analyte	Ion	Ion Source	Competetive Ion Conc. (mg/L)	Adsorption %
Cs^+^	Sr^2+^, Co^2+^	Sr(NO_3_)_2,_ Co(NO_3_)_2_	0	85.65
Cs^+^	Sr^2+^, Co^2+^	Sr(NO_3_)_2,_ Co(NO_3_)_2_	40	65.53
Cs^+^	Sr^2+^	Sr(NO_3_)_2_	40	81,28
Cs^+^	Co^2+^	Co(NO_3_)_2_	40	79,23
