# Supplementary figures and images for: Synthesis of modified nanocomposite material and its use on removal of cesium from aqueous media
Source: Turk J Chem. 2021 Aug 24;46(1):46–58. doi: 10.3906/kim-2105-71 (PMC10734734; doi:10.3906/kim-2105-71)

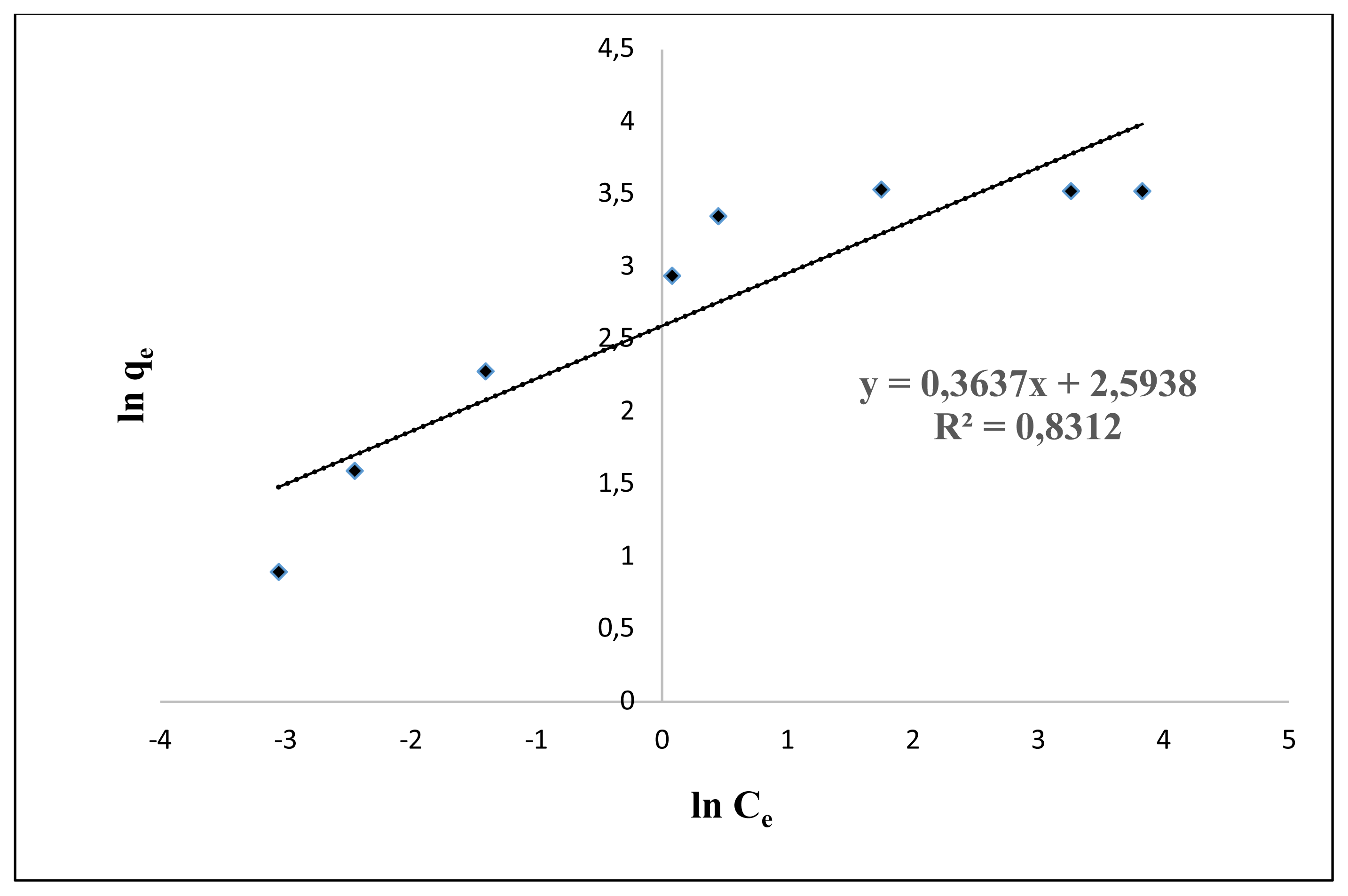

Supplement: Figure S1 — Freundlich isotherm graph obtained from cesium adsorption [file turkjchem-46-1-46s1.tif]

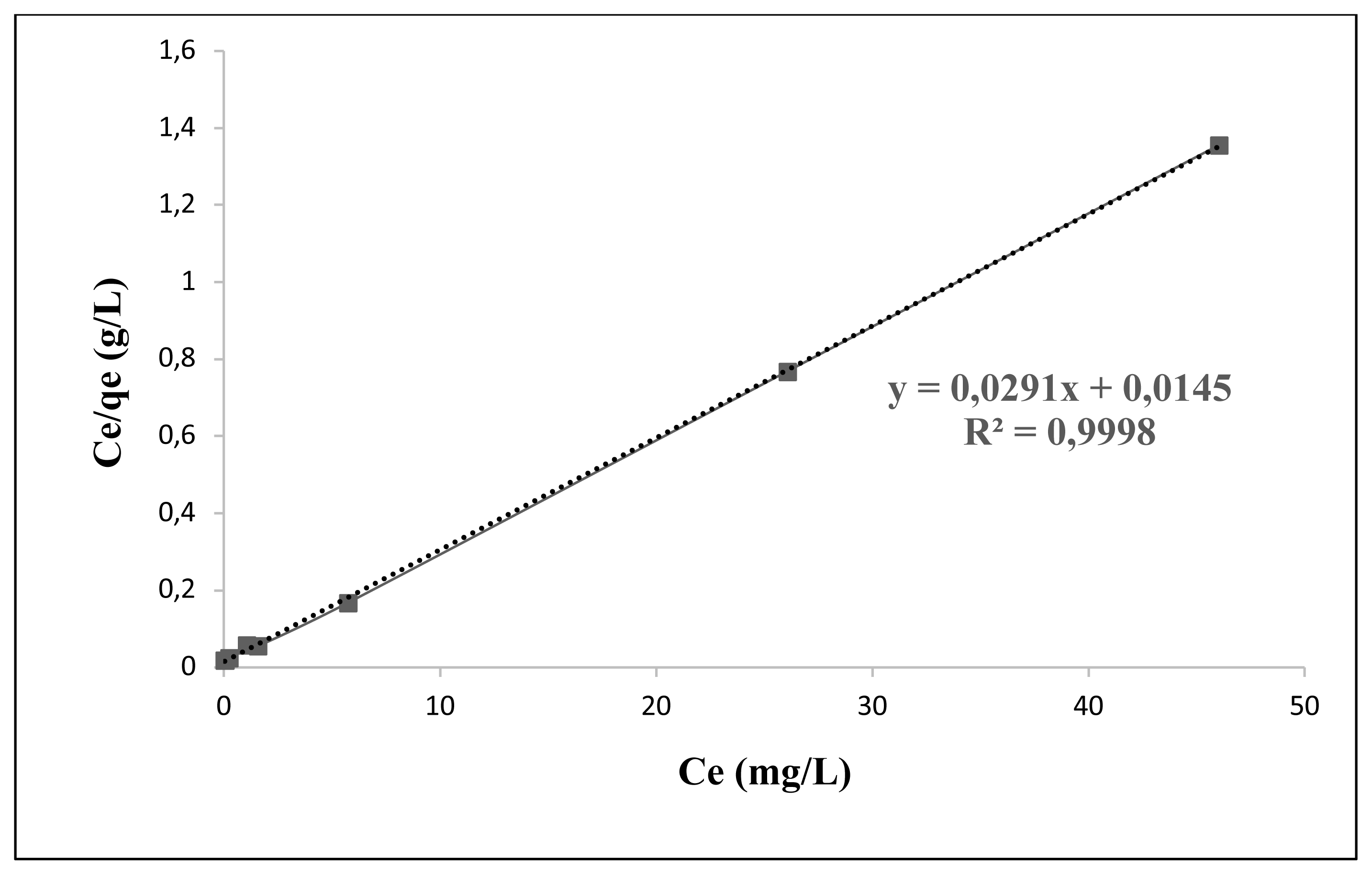

Supplement: Figure S2 — Langmuir isotherm graph obtained from cesium adsorption [file turkjchem-46-1-46s2.tif]
